# The Adjunctive Digital Breast Tomosynthesis in Diagnosis of Breast Cancer

**DOI:** 10.1155/2013/597253

**Published:** 2013-06-17

**Authors:** Tsung-Lung Yang, Huei-Lung Liang, Chen-Pin Chou, Jer-Shyung Huang, Huay-Ben Pan

**Affiliations:** ^1^Department of Radiology, Kaohsiung Veterans General Hospital, Kaohsiung 81362, Taiwan; ^2^National Yang-Ming University, Taipei, Taiwan

## Abstract

*Purpose*. To compare the diagnostic performance of digital breast tomosynthesis (DBT) and digital mammography (DM) for breast cancers. *Materials and Methods*. Fifty-seven female patients with pathologically proved breast cancer were enrolled. Three readers gave a subjective assessment superiority of the index lesions (mass, focal asymmetry, architectural distortion, or calcifications) and a forced BIRADS score, based on DM reading alone and with additional DBT information. The relevance between BIRADS category and index lesions of breast cancer was compared by chi-square test. *Result*. A total of 59 breast cancers were reviewed, including 17 (28.8%) mass lesions, 12 (20.3%) focal asymmetry/density, 6 (10.2%) architecture distortion, 23 (39.0%) calcifications, and 1 (1.7%) intracystic tumor. Combo DBT was perceived to be more informative in 58.8% mass lesions, 83.3% density, 94.4% architecture distortion, and only 11.6% calcifications. As to the forced BIRADS score, 84.4% BIRADS 0 on DM was upgraded to BIRADS 4 or 5 on DBT, whereas only 27.3% BIRADS 4A on DM was upgraded on DBT, as BIRADS 4A lesions were mostly calcifications. A significant *P* value (<0.001) between the BIRADS category and index lesions was noted. *Conclusion*. Adjunctive DBT gives exquisite information for mass lesion, focal asymmetry, and/or architecture distortion to improve the diagnostic performance in mammography.

## 1. Introduction

Breast cancer remains one of the leading causes of death in women over the age of 40 years [1, 2]. Mammography is an effective imaging tool for the detection of early-stage breast cancer, and it is the only screening modality proved to reduce mortality from breast cancer [3–5]. The sensitivity of screening mammography for breast cancer had been reported to be 80%–90% but may be as low as 48% in extremely dense breast [6] because of overlapping dense fibroglandular breast tissue, which substantially reduces the conspicuity of some breast lesions. Digital breast tomosynthesis (DBT) is expected to overcome the inherent limitations of mammography caused by overlapping of normal and pathological tissues during the standard two-dimensional (2D) projections [7–10]. In a DBT system, the X-ray tube moves along an arc during the examination, and a finite number of 2D projections are acquired within a limited angle. The 3D volume of the compressed breast is reconstructed from the 2D projections, allowing enhancement of the information contained in each plane while blurring the off-focus information. Thus, DBT can provide better tissue visualization through the provision of 3D nonoverlapped tissue information. Several studies have shown that tomosynthesis may offer superior diagnostic accuracy, not only in the routine diagnostic practice [9, 11–14], but also in breast cancer screening [15], in the evaluation of breast lesions. Poplack et al. [16] concluded that subjectively, DBT has comparable or superior image quality versus full-field digital mammography (DM) and has the potential to reduce screening recall rates when used in adjunction with DM. Andersson et al. [12] concluded that cancer visibility on DBT in one view is superior to full-field DM in two views and that this would indicate the potential of DBT to increase sensitivity.

 In this study, we compared the diagnostic performance of 59 pathologically proved breast malignancy in a multireader retrospective study to determine whether or not simultaneously viewing DM and DBT is perceived to be more informative in detection (including assessing the features of masses, asymmetries, architectural distortions, and microcalcifications) and diagnosis (BIRADS score) of breast cancers. 

## 2. Materials and Methods

### 2.1. Patients

Inclusion criteria for this study were patients who had pathologically proved breast cancer and had undergone both DM and DBT during the period of January 2012 to November 2012. This retrospective review of research database was conducted with the approval of the institutional review board. The demographic data, including age, clinical symptom and sign, mammographic findings, and histopathologic staging, was recorded as a case report form in a secure research database. The mammographic findings included the density of breast, the type of malignant features (mass, architectural distortion, focal asymmetric density, and calcification), and breast imaging reporting and data system (BIRADS) score. If the findings were mixed, we recorded the most conspicuous findings. In cases where there was more than one lesion, a separate form was created for each lesion.

### 2.2. Image Acquisition

 Selenia Dimensions (Hologic Inc., Bedford, MA) “combo-mode” imaging system with mediolateral oblique and craniocaudal projections was applied in this study with acquiring a traditional digital mammogram and a tomosynthesis scan during the same breast compression. It employs a tungsten (W) target and a selenium (Se) detector with a rhodium (Rh) filter, a silver (Ag) filter for 2D images, an aluminum (Al) filtration in tomosynthesis images. During acquisition, 15 low-dose projection images with exposure parameters of 29 kVp and 44 mAs are obtained over a 15° arc with a continuous exposure method. After acquisition, raw data of the projection images are used for reconstruction to yield images of 1 mm thickness in an orientation paralleling to the detector with totaling 30–80 tomosynthetic images per view depending on the breast thickness being compressed. The reconstructed pixel size is 110–120 *μ*m. The total acquisition time for one breast tomosynthesis view is approximately 3 seconds. Radiation dose to single breast view is about 1.45 mGy. 

 The radiologists viewed individually or sequentially a dynamic cine mode at a mammography workstation (Hologic Inc., SecurView) that included two Barco 5-megapixel monitors (Kortrijk, Belgium), allowing the viewing of one, two, or four images per display for each monitor.

### 2.3. Image Assessment

The selection of cases was performed by one radiologist who did not participate in the subjective rating study and knew from the relevant reports the actual diagnosis of all cases, in particular, the index lesion of interest. The index lesion of interest location was recorded for each case by this radiologist on a data form so that readers knew which lesion to evaluate. Three board-certified radiologists with varying breast imaging experience ranging from 5 to 15 years volunteered for the study. Readers were told to assume the screening DM examination was the woman's baseline examination; hence, no prior DM examinations were provided for comparison. Readers were asked to provide a subjective assessment of how well the combination of DM and DBT examinations is compared with DM alone for the purpose of evaluating the index lesion. A scale of 0 to 2 was provided and used with 0 indicating that DM plus DBT was equivalent or comparable for diagnosis compared with DM, 1 indicating that DM plus DBT was somewhat better for diagnosis, 2 indicating that DM plus DBT was definitely better for diagnosis compared with DM alone. After the interpretation, readers were asked to provide a forced BI-RADS score (1–5) for each index lesion, based on DM reading alone and with additional DBT information. 

### 2.4. Definitions

Based on ACR recommendation [17], type (1–4) of breast density indicates tissue density almost entirely fat, scattered fibroglandular density, heterogeneously dense, and extremely dense, respectively. Focal asymmetric breast density is defined as “asymmetry of tissue density with similar shape on two views but completely lacking borders and the conspicuity of a true mass.”

Architectural distortion is defined as the normal architecture of the breast that is distorted with no definite mass visible. This includes spiculations radiating from a point and focal retraction or distortion at the edge of the parenchyma. Architectural distortion can also be an associated finding. As to the calcifications, amorphous or coarse heterogeneous calcifications are of intermediate concern and fine pleomorphic or fine linear or fine linear branching calcifications are of higher probability of malignancy. Cluster, linear, or segmental distribution of microcalcifications is suspicious for malignancy. Cluster, linear, or segmental distribution of microcalcifications is suspicious for malignancy. 

### 2.5. Data Analysis

We calculate the mean of 57 patient's age. Then, we computed the frequency and proportion of the patient characteristics, the subjective ratings, and the BIRADS ratings of overall readers and cancer cases. For the purpose of this analysis, subjective ratings 1 and 2 were combined. Chi-square test was used to compare the relevance between BIRADS category and index lesions of breast cancer.

## 3. Results 

**Figure 1:**
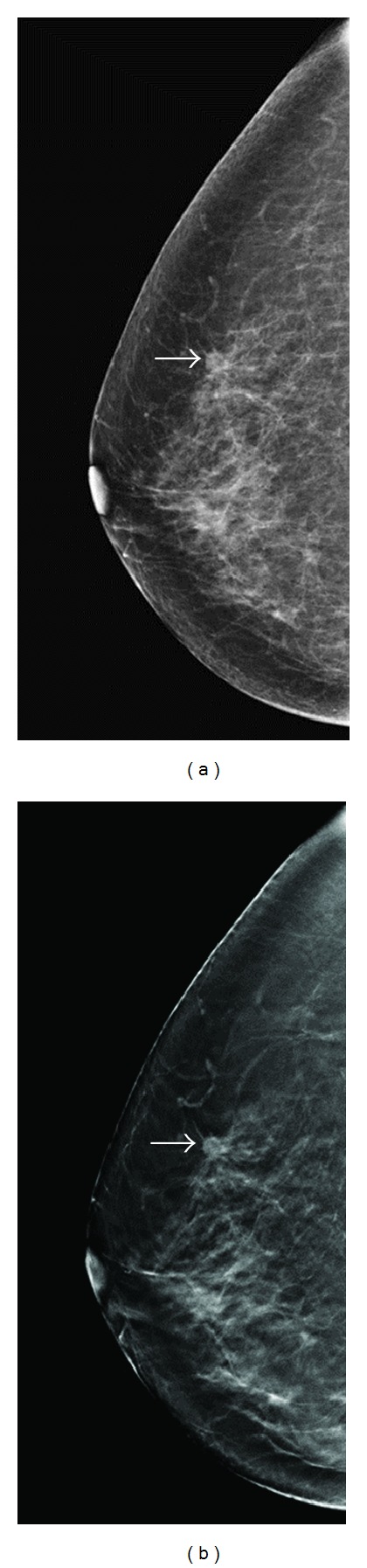
A 66-year-old woman for mammographic screening. (a) Digital mammogram showed a small oval-shaped, well-defined nodule (arrow) over upper-outer quadrant (UOQ) of right breast with BIRADS 0 rated by all the 3 readers. (b) Tomosynthesis revealed a nodule with lobular contour and obviously spiculated margin (arrow). The BIRADS score was rated as 4C by 1 reader and category 5 by 2 readers. The lesion was later proved pathologically to be a breast cancer (T1bN0M0).

**Figure 2:**
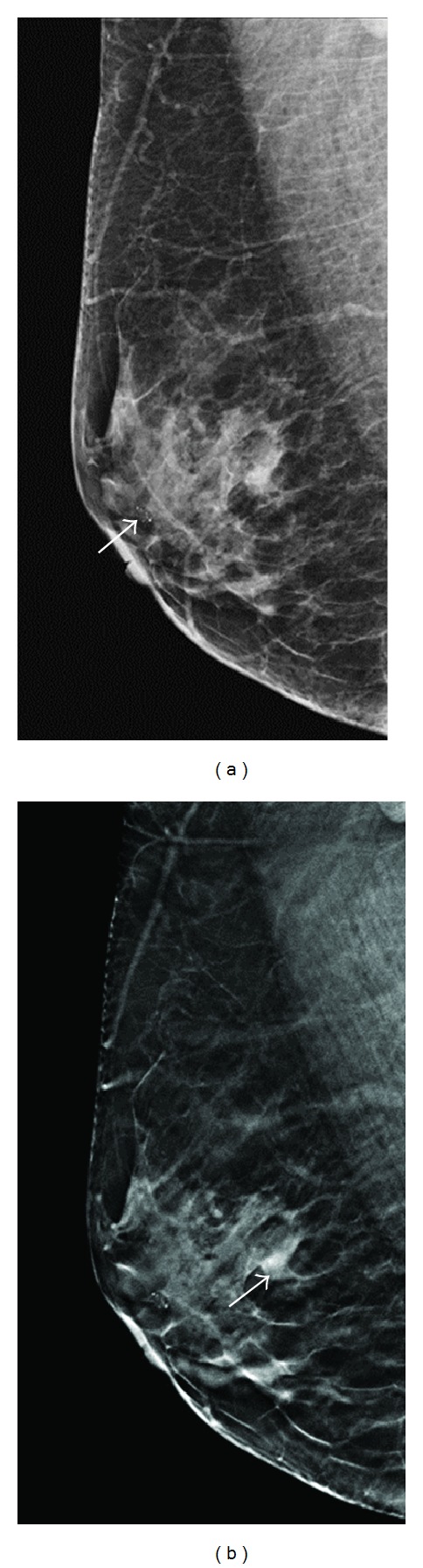
A 57-year-old woman for mammographic screening. (a) Digital mammogram showed a cluster of amorphous microcalcification (arrow) at the subareolar region of right breast, which was proved to be benign in nature by needle biopsy. The initial BIRADS score was rated as 0 by one reader and 4A by two readers. (b) Tomosynthesis revealed a focal asymmetric density around 7 mm in diameter (arrow), which was rated as BIRADS 4B by two readers and 5 by one reader. The lesion was later proved pathologically to be invasive cancer (T1cN0M0).

**Figure 3:**
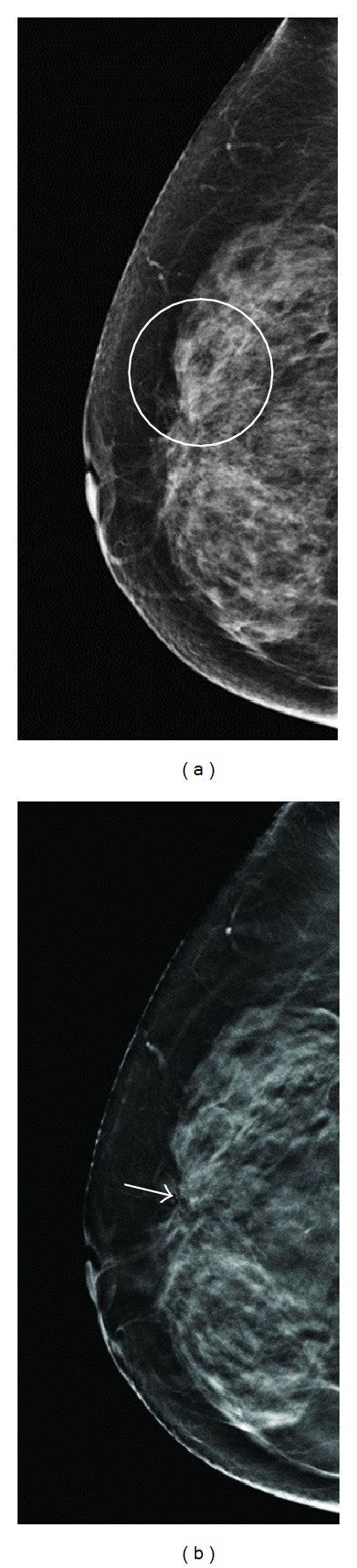
A 59-year-old woman for mammographic screening. (a) Digital mammogram showed focal increased density over UOQ of right breast (circle) with BIRADS rating 0 by all the 3 readers. (b) Tomosynthesis revealed obvious architecture distortion (arrow) with radiating spiculations tethered from retracted tissue. The BIRADS score was rated as 4B by 2 readers and 4C by one reader. Patient received partial mastectomy with pathologically proved intraductal cancer, intermediated type (TisN0M0).

**Figure 4:**
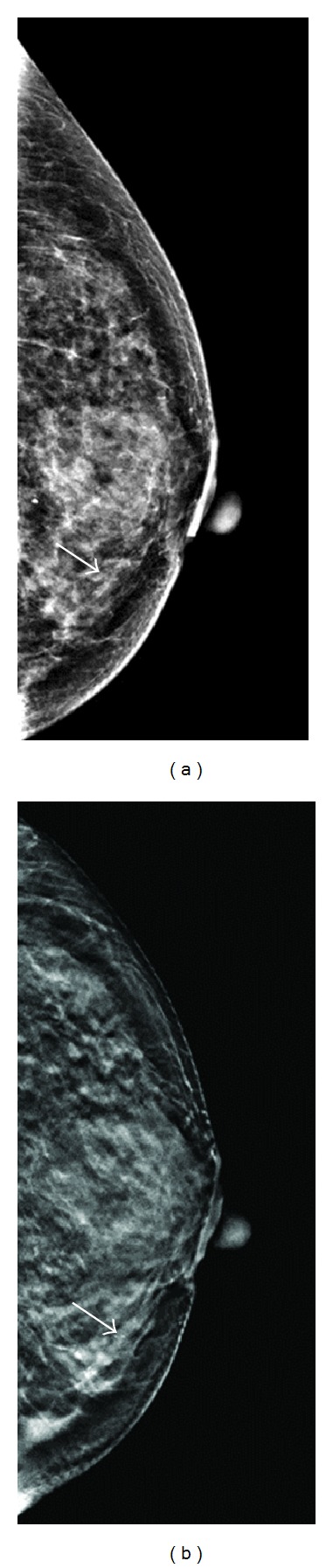
A 55-year-old female had partial mastectomy 5 years ago due to intraductal carcinoma in situ in right breast with yearly mammographic follow up. (a) Digital mammogram showed microcalcifications (arrow) with segmental distribution over the lower-inner quarter of the left breast. The BIRADS score was rated as 0 by two readers and 4A by one reader. (b) Tomosynthesis revealed the microcalcifications being inside the dilated tubular structure, which was toward the nipple. The BIRADS score was upgraded to 4B by one reader and 4C by two readers. The lesion was proved pathologically to be an invasive cancer (T1cN0M0).

**Figure 5:**
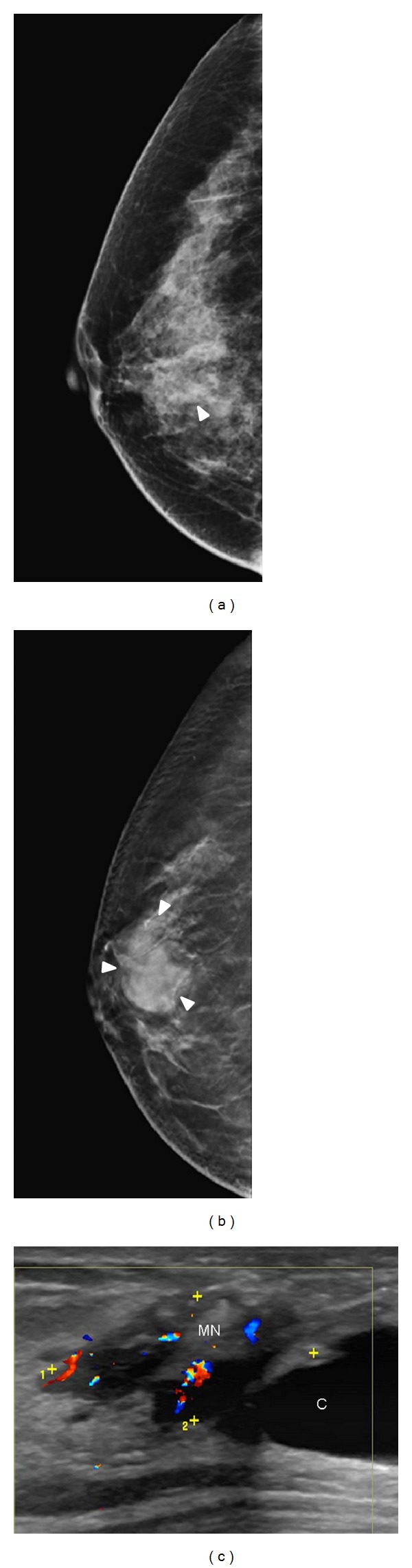
A 61-year-old woman with palpable mass in the right breast. (a) Digital mammograms showed focal increased density (arrowhead) without architectural distortion in the right breast. (b) Tomosynthesis clearly delineated the smooth border of a mass lesion (arrowhead) without showing malignant feature. This case was considered as true negative in both digital mammography and tomosynthesis. (c) Color ultrasound showed a cystic-like lesion (C) with hypervascular mural nodule (MN). The lesion was proved to be ductal carcinoma in situ, TisN0M0.

Fifty-seven patients (mean age 53.5 years, range 26–89 years) with pathologically proved breast cancers who had undergone combo DBT for either screening or diagnostic purposes were enrolled in this retrospective study. Two patients had a second malignancy in the ipsilateral breast. Thus, a total of 59 breast cancers were reviewed in this study. Thirty patients (52.6%) were symptomatic with positive breast physical examination during mammography taken. The location of the breast cancers were 31 lesions on the right and 28 on the left. As to the breast composition (BI-RADS type), most (up to 79%) patients in our series had dense breast (type 3 or 4) with type 1 in two (3.5%) patients, type 2 in 10 (17.5%), type 3 in 27 (47.3%), and type 4 in 18 (31.6%). Of the 59 index lesions, 17 (28.8%) lesions were presented as mass (Figure 1), 12 (20.3%) as focal asymmetry/density (Figure 2), 6 (10.2%) as architecture distortion (Figure 3), and 23 (39.0%) as calcifications (Figure 4). One intracystic tumor (1.7%), which was clinically palpable and diagnosed by US images, failed to show malignant feature on either DM or tomosynthesis images (Figure 5) and therefore was considered as truly false negative. BIRADS category of the fifty-seven patients was initially rated as 0 in 20 cases (35%), 4A in 8 (14%), 4B in 9 (15.8%), 4C in 7 (12%), and category 5 in 13 cases (22.8%), respectively, with final clinical staging of ductal carcinoma in situ (DCIS) in 16 cases (28.1%), stage 1 in 17 cases (29.8%), and T1N1 or above in 24 cases (42.1%). The demographic and clinical results of these 57 patients were listed in Table 1.

 For the overall 59 target lesions interpreted by 3 readers, combo DBT was perceived to be more informative for diagnosis in 48% (85/177) of the subjective ratings (59 lesions × 3 readers = 177 ratings). A superior rating of the index lesions was considered in 30 of 51 (58.8%) mass lesions, 30 of 36 (83.3%) density lesions, 17 of 18 (94.4%) architecture distortion lesions, while only 8 of 69 (11.6%) in calcification lesions. The 85 superior ratings of 1 or 2 occurred in 34 patients in which at least one radiologist had given a positive rating. Of whom, 73.5% were dense breasts (type 3 or 4) with type 1 breast in 2 (5.9%) patients, type 2 breast in 7 (20.6%) patients, type 3 breast in 16 (47%) patients, and type 4 breast in 9 (26.5%) patients. The ratings of overall and each relevant index lesion were listed in Table 2.

As to the retrospective review of BI-RADS score in the 57 cancer patients (171 ratings), BI-RADS 0 and 4A were rated 64 (37.4%) and 33 (19.3%) on DM versus 10 (5.8%) and 29 (16.9%) on combo tomosynthesis, respectively. Of the 64 BIRADS 0 rated on DM, 10 (15.6%) ratings were still categorized as BIRADS 0 on combo tomosynthesis, while upgraded to 4A in 4 (6.3%), 4B in 22 (34.4%), 4C in 17 (26.6%), and category 5 in 11 (17.2%), whereas, in the 33 BIRADS 4A ratings on DM, 24 (72.7%) were still categorized the same, while being upgraded to 4B in 5 (15.2%) and 4C in 4 (12.1%), respectively, with additional reviewing of the 3D tomosynthesis images. Of the BIRADS 4A patient group, calcification was the dominant lesion in 31 (93.9%) of the 33 ratings. The overall forced BI-RADS scores on digital mammograms and tomosynthesis were listed in Table 3. Although there seemed to be of little improvement of the DBT diagnosis on calcification lesions, markedly improved diagnostic performance of density, distortion, and mass lesions was noted in this study. The forced BIRADS score of 0, 4, and 5 versus each type of lesion was listed in Table 4. Comparing the BIRADS category (category 0 versus 4A versus 4B + 4C + 5) and index lesions by the use of chi-square test, a significant *P* value  (<0.001) was noted (Table 5). 

## 4. Discussion

The malignant features of breast cancers can be classified as mass, focal asymmetry, architecture distortion, and microcalcification. Better delineations of the lesion border and margin result in a more definitive interpretation. Previous studies concluded that the shape and margin of the mass in tomosynthesis were well characterized than DM [9, 13]. Thus, small undulating contour or subtle speculated margins of masses can be identified on a thin slide without normal breast tissue masking. Our study confirmed that 58.8% of mass index lesions had superior rating on tomosynthesis versus DM alone. 

Focal asymmetric breast density is found in approximately 3% of mammograms [18]. A review of the literature showed that malignancy can be found in 0%–14% of asymmetric breast tissue biopsies, and any associated features of possible malignancy, or a clinically palpable mass mandates tissue diagnosis [19]. However, the lesion presented as focal asymmetric breast density on 2D mammograms may lack its conspicuous borders, making the diagnosis of malignancy difficult in a sole “asymmetry” finding. Although, the focal density on DBT is presented as an ill-defined mass but it still definitely has a border and volume size. Thereafter, it can be easily distinguished from an island of normal breast tissue. In other words, a lesion with 5 mm in size would be detected in at least five 1 mm contiguous slices even if it presented as a focal asymmetry. In our study, 83.3% density reading were rated as superior to that of DM only with 85.2% (23/27) BIRADS 0 being upgraded to BIRADS 4 or 5.

 On mammograms, the breast is seen as a directionally oriented-textured image due to the presence of several piecewise linear structures such as ligaments, ducts, and blood vessels. The presence of tumor, inflammation, trauma, or surgery may change the orientation of normal architecture, whereas the presence of overlapping dense fibroglandular breast tissue may substantially reduce the conspicuity of the changes. It was reported that architectural distortion accounted for 12% to 45% of overlooked or misinterpreted breast cancer cases in screening mammographies [20, 21], and it constituted the most commonly missed abnormality in false-negative cases. As the barrier of false negative for distortion lesions is chiefly related to overlapped tissue, DBT was proved to be an expectant method to solve this problem [14]. In our study, 94.4% distortion reading were rated as superior to that of DM only with all the BIRADS 0 being upgraded to BIRADS 4 or 5.

 Our study also showed markedly improved diagnostic accuracy for noncalcified lesions on tomosynthesis mammograms with 78.2% initially scored as BIRADS 0 on DM being upgraded to equal or higher than RIRADS 4B, which may allow for the replacement of conventional supplemental mammographic views. Our series showed only mild-modest improvement of the diagnostic accuracy in the patient group initially scored as BIRADS 4A with 72.7% of patients remaining with the same score. It was because most of the lesions (93.9%) in this patient group were microcalcifications. The clinical benefits of tomosynthesis on calcified lesions may be still debated with the concern of not depicting calcifications as well as traditional mammography [16]. However, increasing the slice thickness would increase the ability to perceive a 3D configuration of calcifications, and the extents of accompanying microcalcifications may be better depicted on BDT than on 2D mammograms [22]. Thereafter, Spangler et al. [22] concluded that DM appeared to be slightly more sensitive than DBT for the detection of calcification (84% versus 75%). However, diagnostic performance as measured by area under the curve using BIRADS was not significantly different. Other studies had also supported the diagnostic performance of digital breast tomosynthesis in conjunction with [23] or independently [24, 25] of full-field DM. 

 A major limitation of this study was that it involved a nonblinded retrospective review of only pathologically proved breast cancer images. The true diagnostic accuracy of combo DBT in general population is unclear. But with more familiarity with the imaging features of breast malignancy on DBT, it may help us interpret the tomosynthesis more precisely. In addition, the term of focal asymmetry is defined in 2D side by side interpretation, but for ease to compare with previous tomosynthesis studies, we still use the term of focal asymmetry instead of focal density.

In conclusion, adjunctive DBT gives exquisite information for mass lesion, focal asymmetry, and/or architecture distortion to improve the diagnosis in mammography with comparable performance to the pattern of microcalcifications on 2D mammogram, yet the additional associating findings such as intraductal calcification may give some clues to confirm malignancy. 

## Figures and Tables

**Table 1:** Demographic data of the 57 breast cancer patients.

	Patients = 57	
Patient characteristics	*N *	%
Age (mean)	53.7	
Symptom/sign: Y/N	30/27	52.6/47.4
Part: L/R	27/30	47.4/52.6
Type		
1	2	3.5
2	10	17.5
3	27	47.4
4	18	31.6
Characteristic		
Mass	17	28.8
Density	12	20.3
Distortion	6	10.2
Calcifications	23	39.0
None	1	1.7
BI-RADS		
0	20	35.1
4A	8	14.0
4B	9	15.8
4C	7	12.3
5	13	22.8
TNM stage		
Tis + T1mi	16	28.1
T1N0	17	29.8
T1N1 or above	24	42.1

**Table 2:** The results of rating in each type of lesions and overall lesions.

	Calcification		Density		Distortion		Mass		None		Overall	
Rating	*n *	%	*n *	%	*n *	%	*n *	%	*n *	%	*n *	%
0	61	88.4	6	16.6	1	5.5	21	41.2	3	100	92	51.98
1	4	5.8	15	41.7	5	27.8	24	47	0	0	48	27.12
2	4	5.8	15	41.7	12	66.7	6	11.8	0	0	37	20.90

	69		36		18		51		3		177	

0: equal; 1: somewhat better; 2: definitely better.

**Table 3:** The overall forced BI-RADS score on digital mammograms and tomosynthesis.

3D	2D							
	2	3	0	4A	4B	4C	5	All (rating)
2	2	0	0	0	0	0	0	2
3	0	0	0	0	0	0	0	0
0	0	0	10	0	0	0	0	10
4A	0	1	4	24	0	0	0	29
4B	0	1	22	5	12	0	0	40
4C	1	0	17	4	13	7	0	42
5	0	0	11	0	8	13	16	48

All	3	2	64	33	33	20	16	171

**Table 4:** The forced BI-RADS score (0,4, 5) of each type of lesions on digital mammograms and tomosynthesis.

3D	2D												Total
	Cal			Density			Distortion			Mass		
	0	4	5	0	4	5	0	4	5	0	4	5
BI-RADS													
0	4	0	0	4	0	0	0	0	0	0	0	0	8
4	5	54	0	21	3	0	10	0	0	7	8	0	108
5	2	0	4	2	2	1	2	2	0	5	16	12	48

Total	11	54	4	27	5	1	12	2	0	12	24	12	164

**Table 5:** Comparison of BIRADS score and lesions type.

Lesion	BIRADS (3D)			Total
	0	4A	4B, 4C, 5	
Calcification	4	26	39	69
Density	4	3	28	35
Distortion	0	0	15	15
Mass	0	0	48	48

Total	8	29	130	167

*P* value < 0.001 (chi-square test).
